# Editorial: Equity, diversity, and inclusion in rehabilitation sciences

**DOI:** 10.3389/fresc.2023.1334054

**Published:** 2023-11-23

**Authors:** Adria Quigley, Hellen Myezwa, Caitlin McArthur

**Affiliations:** ^1^ School of Physiotherapy, Dalhousie University, Halifax, NS, Canada; ^2^ Acquired Brain Injury, Nova Scotia Health Authority, Halifax, NS, Canada; ^3^ School of Therapeutic Sciences, University of the Witwatersrand, Johannesburg, South Africa

**Keywords:** equity, diversity & inclusion, rehabilitation, physiotherapy, occupational therapy, anti-racism, intersectionality

**Editorial on the Research Topic** Equity, diversity, and inclusion in rehabilitation sciences

Equity, Diversity, and Inclusion have become critical issues in the field of rehabilitation sciences (1). Like many other professions, physiotherapists, occupational therapists and other rehabilitation providers are reckoning with the ongoing impacts of colonialism, racism, stigmatization, and discrimination that form part of the fabric of our health care systems (2–10). Now, perhaps more than ever, there is a demand for equitable and timely access to rehabilitation. Incorporating principles of equity, diversity, inclusion, and accessibility into education, clinical practice, and research are essential for addressing the unique needs of rehabilitation clients. There is also a call to integrate anti-oppressive and anti-racist frameworks into rehabilitation care. Strategies that promote accessible and affordable participation, health promotion, technology, and interdisciplinary collaboration in rehabilitation are needed. Intersectionality is a concept that highlights how various aspects of an individual's identity (such as race, gender, sexuality, class and disability) can intersect and interact to create unique experiences of discrimination, advantage or disadvantage, or privilege (11). Within the context of rehabilitation science, we need to understand these concepts and illuminate how to better serve equity-deserving groups.

We are pleased to serve as guest associate editors for the Research Topic: Equity, Diversity, and Inclusion in Rehabilitation Sciences. While it is not possible to address all aspects of Equity, Diversity, and Inclusion in one issue, this Research Topic may be thought of as the beginning of the conversation. What is missing from the Research Topic is an examination of the impact of gender, sexual orientation, race and religion, and socioeconomic status on rehabilitation service delivery and outcomes. We hope that this Research Topic will inspire focus on these areas as they pertain to rehabilitation.

We are fortunate to have nine articles included in this Research Topic, five of which are original research articles (Jiancaro et al., Sekome et al., Ibáñez-Carrasco et al., Chumo et al., Claus et al.), two are perspective articles (Lurch et al., Dorsey Holliman et al.), and two are brief research reports (Fricke et al., Oancea-Matei et al.). From these nine articles, two main themes emerged. The first theme is *the importance of knowledge transfer of Equity, Diversity, and Inclusion principles* within the rehabilitation sciences professions. Lurch and colleagues provided an introduction to African philosophies and how these ways of thinking may challenge Western biomedical approaches to health care (Lurch et al.). The authors argue that the default physiotherapy identity, curricula, and practices are derived from Western epistemologies which have devalued other ways of knowing. Lurch et al. describe African philosophies that can benefit rehabilitation providers and their clients across the world. Fricke et al. provide an assessment of the impact of an anti-racism simulation workshop among rehabilitation providers as a strategy to address racial microaggressions toward Indigenous peoples in the workplace. As part of this study, the authors asked workshop participants to fill in a post-workshop survey. Four themes emerged from the participant surveys: so much to unlearn, remain humble, resist the silence, and discomfort is okay. Sekome et al. describe undergraduate physiotherapy training in South Africa aimed at providing equitable services for disadvantaged rural and urban communities. The study emphasizes the theoretical and classroom-based learning experiences of physiotherapy students and propose that students can become agents of change to expand access to rehabilitation services for equity-deserving communities. Ibáñez-Carrasco et al. describe the development, implementation, and evaluation of a community of practice (HIV in Motion) aimed at advancing rehabilitation interventions for people living with HIV. The community of practice consisting of people living with HIV, researchers, representatives from non-profit organizations, exercise personnel, trainees, and health professionals had a total of 451 participants and 72% reported connecting with another participant because of the 8 online sessions. Outcomes of this study included sharing the lived experiences of people living with HIV, the inclusion of diverse voices, and the mentorship of peer-researchers. These four articles highlighted ways in which knowledge transfer has begun in the area of equity, diversity, and inclusion in rehabilitation sciences and also identify opportunities for ongoing work.

The second theme that emerged from the articles was *identifying the attitudes, needs, and barriers of equity-deserving groups*. Dorsey Holliman et al. used the 2018 National Health Interview Survey to assess the unmet healthcare needs of Black and Hispanic adults with disabilities. Their study revealed that Black and Hispanic individuals living with disabilities experienced greater healthcare access disparities than Black and Hispanic adults without disabilities. Their findings also indicated that almost 30% of Black and Hispanic adults with disabilities forewent healthcare services due to cost compared to individuals with no disabilities. Claus et al. described the needs and social determinants of health of racially and ethnically diverse uninsured patients with rehabilitation diagnoses during the COVID-19 pandemic. The authors reported medical issues, equipment needs, and mental health concerns as the top three needs identified by participants. Another study investigated attitudes of Romanian participants toward people with bionic eyes and limbs, cochlear implants, and people with disabilities as represented by character vignettes (Oancea-Matei et al.), revealing that personalities that were more agreeable, extraverted, open-minded, intellectually complex, and less neurotic were associated with more positive attitudes to disability*.* Interestingly, the most negative attitudes were directed toward the character vignette with a bionic eye, suggesting that more work should be done to educate individuals about bionic devices. Jiancaro et al. investigated factors influencing implementation of a pilot online community-based exercise intervention for people living with HIV, identifying 55 factors influencing implementation spanning natural, societal, organizational, personal and human factors. Chumo et al. conducted interviews with employers, entrepreneurs, and officials in Kenya to find out how to socially support employed people with disabilities in informal settlements. Participants reported that inadequate or poorly administrated resources, work dissatisfaction, and workplace conflict that contributed to non-inclusion were challenges to social support for people with disabilities. Clearly there is much work to be done in empowering equity-deserving groups in the field of rehabilitation.

This Research Topic highlights the advances in our collective knowledge and advocacy that are already taking place in this field. Many of the articles offer strategies to change rehabilitation professions for the better, while others suggest priorities for further research. We hope that rehabilitation science practitioners, trainees, and researchers feel inspired to have brave conversations moving forward.
